# Emerging Targeted Therapies in Advanced Non-Small-Cell Lung Cancer

**DOI:** 10.3390/cancers15112899

**Published:** 2023-05-24

**Authors:** Shenduo Li, Guilherme Sacchi de Camargo Correia, Jing Wang, Rami Manochakian, Yujie Zhao, Yanyan Lou

**Affiliations:** 1 Division of Hematology and Medical Oncology, Mayo Clinic, 4500 San Pablo Rd, Jacksonville, FL 32224, USAcorreia.guilherme@mayo.edu (G.S.d.C.C.);; 2 Department of Medicine, Mayo Clinic, 4500 San Pablo Rd, Jacksonville, FL 32224, USA

**Keywords:** NSCLC, targeted therapy, phase I/II, clinical trial, first-in-human, EGFR

## Abstract

**Simple Summary:**

The discovery of actionable oncogenic driver mutations in non-small-cell lung cancer (NSCLC) has revolutionized the treatment and prognosis of this dreadful disease. New targeted therapies are being developed and tested in early phase clinical trials rapidly. We summarize emerging first-in-human clinical trials of targeted therapy that have been conducted or initiated in the past year. We hope this review will benefit our readers to be familiar with new advances in this field and create more opportunities for eligible patients to access clinical trials.

**Abstract:**

Lung cancer remains the leading cause of cancer-related mortality worldwide. Non-small-cell lung cancer (NSCLC) is the most common type and is still incurable for most patients at the advanced stage. Targeted therapy is an effective treatment that has significantly improved survival in NSCLC patients with actionable mutations. However, therapy resistance occurs widely among patients leading to disease progression. In addition, many oncogenic driver mutations in NSCLC still lack targeted agents. New drugs are being developed and tested in clinical trials to overcome these challenges. This review aims to summarize emerging targeted therapy that have been conducted or initiated through first-in-human clinical trials in the past year.

## 1. Background

Lung cancer is the second-most diagnosed cancer and the leading cause of cancer death worldwide [1]. Non-small-cell lung cancer (NSCLC) accounts for 85% of all lung cancer cases. Among NSCLC, the most common histological subtype is adenocarcinoma (LUAD), followed by squamous cell carcinoma (LSCC). For NSCLC patients at an advanced stage, tumor molecular profiling and biomarker testing are widely adopted as the standard of care that leads to a more individualized treatment plan tailored from multiple options including chemotherapy, immune checkpoint inhibitors, and targeted therapy. Targeted therapies, which are drugs interfering with tumor-specific molecules, are only suitable for NSCLC patients who are found to have actionable mutations by tumor genetic profiling, most commonly next-generation sequencing (NGS). With the advanced technique and decreased cost of NGS, all patients at advanced stages are recommended to test for tumor genetic alterations performed on the tumor tissue or by examining circulating tumor DNA (ctDNA). So far, as of the time of this publication, the following genetic driver alterations have targeted agents approved by the U.S Food and Drug Administration (FDA): EGFR mutations, ALK rearrangements, ROS1 rearrangements, RET rearrangements, NTRK fusions, MET exon 14 skipping mutation, KRAS G12C mutation, BRAF V600E mutation, and ERBB2 (HER2) mutation [2,3,4]. Newer agents are being developed for these molecular targets aiming to induce a more profound response and overcome treatment resistance. Additional translational studies are exploring novel driver mutations, turning traditionally undruggable mutations into therapeutic targets. Furthermore, common tumorigenic signaling pathways such as PI3K/AKT/mTOR and RAF/MEK/ERK have been the focus of targeted drug development for several years. Targeting such pathways along with tumor cell surface receptors by using combination therapy is a promising strategy.

**Figure 1:**
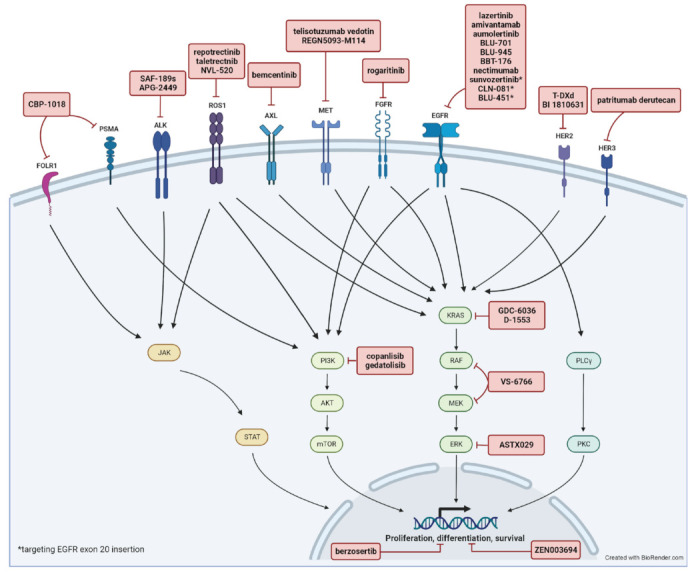
Summary of molecular targets and involved pathways.

In this review, we focus on new investigational targeted agents pipelined into human clinical trials for advanced NSCLC in the past year including trials for advanced LSCC. This is an update from a prior review of early-phase clinical trials on targeted therapy published in 2021 [4]. We identified references by searching the American Society of Clinical Oncology (ASCO) annual meeting abstracts, the World Conference on Lung Cancer (WCLC) abstracts, and information from clinicaltrials.gov over the past year. We only included first-in-human, phase I/II clinical trials in advanced/metastatic NSCLC. A total of 20 phase I, 16 phase I/II, and 10 phase II clinical trials were included in this review. A summary of included trials is shown in Table 1, Table 2 and Table 3 with a summary of molecular targets and involved pathways depicted in Figure 1. Each trial was individually reviewed. We excluded pre-clinical studies and phase III clinical trials. Other emerging systemic therapeutic strategies such as immune checkpoint inhibitors or adoptive cell therapy are beyond the scope of this review.

## 2. EGFR

Epidermal growth factor receptor (EGFR) mutations are found in 15–20% of NSCLC in Western populations and in 45–50% of NSCLC in Asian populations [5]. The most common mutations are deletions in exon 19 and L858R point mutation in exon 21. EGFR tyrosine kinase inhibitors (TKI) achieved great success in treating patients with advanced NSCLC harboring sensitizing EGFR mutations in the last decade. Osimertinib, a third-generation EGFR TKI, has become the first-line therapy for those patients including patients who carry T790M mutation resistant to first/second-generation TKIs. However, resistance to osimertinib inevitably occurs, leading to disease progression. For those patients who progress on osimertinib, chemotherapy remains as the subsequent therapy option unless enrolling in clinical trials. In addition to the lack of further effective targeted therapy, the value of immunotherapy in those patients is questionable. In a small subgroup analysis from the Impower150 trial, the combination of atezolizumab, bevacizumab, carboplatin, and paclitaxel (ABCP) was shown to have a survival benefit in patients with sensitizing EGFR mutations who had disease progression on TKI [6]. Whereas in a recent retrospective study, adding immunotherapy alone to chemotherapy is associated with a worse overall survival [7]. To overcome treatment resistance and develop more effective targeted therapies beyond osimertinib, recent early phase clinical trials focus on the following strategies: new EGFR TKI that are active against resistance mutations such as T790M or C797X, duo-blockade using two EGFR inhibitors, combine EGFR TKI with another targeted agent. Table 1 summarizes recent first-in-human clinical trials of EGFR targeted agents.

Lazertinib is a brain-penetrant EGFR TKI that targets typical EGFR mutations and T790M mutations but spares wild-type EGFR [8]. It was tested in a phase I/II trial of 127 patients with EGFR T790M-mutant NSCLC who had disease progression after TKI therapy (NCT03046992). The trial has an impressive ORR of 54% [9,10]. In another phase I trial, Lazertinib was assessed in the front-line setting for EGFR-mutant NSCLC patients and showed a favorable efficacy. The overall response rate (ORR) was 70% and the median progression-free survival (PFS) was 24.6 months in the optimal 240mg dosing cohort that contained 43 patients [11]. A phase III, randomized, double-blind trial (LASER301) is currently open to evaluate Lazertinib vs. gefitinib in the first-line setting of EGFR-mutant NSCLC treatment (NCT04248829).

Amivantamab is an EGFR-MET bispecific antibody that was approved to treat NSCLC with EGFR exon 20 insertions. The combination of amivantamab and lazertinib, targeting EGFR at both its extracellular and catalytic domains, was shown to have synergistic inhibition of tumor growth in preclinical [12]. This combination was recently evaluated in patients with EGFR-mutant NSCLC who had disease progression on EGFR TKI in the phase I CHRYSALIS-2 trial. The trial enrolled 116 patients who were heavily pre-treated (median prior lines of therapy was 3), and showed an encouraging response rate of 32% [13]. An updated result from cohort A that contains patients who had disease progression on osimertinib and chemotherapy was presented at ASCO 2022 [14]. The ORR was 33% with a median duration of response (DoR) of 9.6 months. The toxicity profile is manageable, and grade 3 or above treatment-related adverse events (TRAEs) include infusion-related reactions (7%), acneiform dermatitis (5%), and hypoalbuminemia (4%) 2022 [14]. In the first-line setting, amivantamab plus lazertinib showed an ORR of 100% in 20 treatment-naïve patients who harbored EGFR exon 19 deletion or exon 21 L858R 2022 [15]. At a median follow-up of 22.3 months, the median DoR and PFS were not reached. The toxicity was consistent with previous trials [15]. The combination of amivantamab and lazertinib has demonstrated encouraging anti-tumor efficacy in both osimertinib-resistant and treatment-naïve subgroups of NSCLC patients in early phase clinical trials. The final results of those trials will be important to show whether there is a consistent trend. Moving forward, a phase III, randomized trial (MARIPOSA) is designed to compare the combination of amivantamab and lazertinib to osimertinib monotherapy as the first-line treatment for EGFR-mutant NSCLC [16]. It aims to enroll 1074 patients worldwide. Notably, this trial only includes patients with EGFR exon 19 deletion or L858R substitution, but not other less common mutations.

Aumolertinib is a third-generation EGFR TKI shown to be effective against T790M mutation. In a phase II APOLLO trial, 244 patients with T790M positive NSCLC who had disease progression on first- or second-generation EGFR TKIs received aumolertinib. The ORR was 68.9% and the median PFS was 12.4 months. The efficacy sustained in patients with CNS metastasis [17]. Icotinib is a first-generation EGFR TKI that is effective against CNS metastasis [18]. The combination of aumolertinib and icotinib is being tested as first-line therapy in NSCLC patients with brain metastasis in a phase I/II trial in China (ChiCTR2100044216). A total of 12 patients with brain metastasis were enrolled in the phase I arm. The ORR was 100%, and no grade 4 or 5 AEs were observed [19]. Long-term outcomes are yet to be determined and the phase II arm of the trial is undergoing.

BLU-945 is a next-generation EGFR tyrosine kinase inhibitor (TKI) that is highly selective for on-target resistance EGFR mutations such as C797S and T790M, while sparing EGFR wildtype. Thus, it is potentially to be used in combination with other EGFR TKI to increase antitumor efficacy without increasing toxicity. BLU-945 has antitumor activity including intracranial response in osimertinib-resistant patient-derived xenograft (PDX) models [20]. It is currently being evaluated in the SYMPHONY trial (NCT04862780)–a phase I/II trial for patients with EGFR mutations who had received at least one EGFR TKI. Patients will receive either BLU-945 monotherapy or in combination with osimertinib. The primary endpoints are maximum tolerated dose, recommended phase II dose, safety in the phase I study, and overall response rate in the phase II study [21]. This trial is currently open for enrollment. The final results will help assess the role of BLU-945 in overcoming EGFR resistance and the potential clinical application of dual EGFR inhibitors.

BBT-176 is a 4th generation EGFR TKI with high potency against C797S mutation. In a recent phase I trial (NCT04820023), 25 patients with EGFR-mutant NSCLC who had cancer progression on at least one EGFR TKI were enrolled. Most of them (64%) received three or more lines of therapy. A total of 32% of them were found to have C797S mutation. TRAEs were mostly low grade including GI toxicities, dizziness, and platelet count decrease. Two patients with tumors harboring triple EGFR mutations of exon 19 del/T790M/C797S showed tumor shrinkage in both target and non-target lesions [22]. The trial is ongoing to further optimize BBT-176 dosing and evaluate clinical efficacy.

BLU-701 is a brain-penetrant EGFR TKI with high potency on common EGFR mutations plus C797X resistance mutations, while sparing wild-type EGFR [23]. It is being evaluated in a phase I/II first-in-human study (HARMONY trial) for patient with EGFR-mutant NSCLC. The phase I arm has three parts: 1A, 1B, and 1C, which are testing BLU-701 alone, BLU-701 plus osimertinib, and BLU-701 plus carboplatin and pemetrexed, respectively. The phase II arm will be using the recommended phase 2 dose (RP2D) from phase I result, and has an designated group (part 2A) for NSCLC patients harboring EGFR C797X mutation [23]. The trial is currently recruiting and expected to finish in 2024.

Patritumab deruxtecan is an antibody-drug conjugate consisting of an anti-HER3 antibody attached to a topoisomerase I inhibitor payload. It demonstrated clinical efficacy in treating advanced EGFR-mutant NSCLC after failure of EGFR TKI in a phase I trial (NCT03260491) [24]. The phase II and phase III trials, namely HERTHENA-Lung01 (NCT04619004) and HERTHENA-Lung02 (NCT05338970), are actively recruiting to further confirm the efficacy and safety of patritumab deruxtecan in patients with EGFR-mutant NSCLC with disease progression on EGFR TKI. Additionally, Patritumab deruxtecan is also being tested for treating NSCLC with wild-type EGFR in one arm of the above phase I trial. A total of 47 patients who had received prior chemotherapy with or without immunotherapy were treated with patritumab deruxtecan. The ORR was 28% and the median PFS was 5.4 months [25]. Notably, in 17 patients with driver genomic alterations, 35% achieved objective response. The most common grade ≥ 3 TEAEs were neutropenia (26%), thrombocytopenia (15%), and fatigue (15%), and drug-related interstitial lung disease occurred in 4 pts (9%; 0 grade ≥ 3) [25].

## 3. EGFR TKI in Combination with Other Targeted Agents

Anlotinib is a multi-targeting tyrosine kinase inhibitor (TKI) that has anti-tumor activity in several solid tumors [26]. Adding anlotinib to osimertinib was able to overcome treatment resistance in EGFR T790M-positive NSCLC in an in vitro study and a retrospective clinical study [27,28]. This combination was recently tested in a first-line setting for NSCLC in a phase I/IIa trial (NCT04770688). A total of 25 treatment-naïve patients with EGFR-mutant NSCLC were treated with anlotinib plus osimertinib [29]. The ORR was 65.2% and DCR was 95.7%. Median PFS was not reported. Grade 3 or higher AEs occurred in 20% patients, and the most common AEs were platelet count decreased (56.5%), thyroid-stimulating hormones increased (39.1%) and diarrhea (30.4%) [29]. Osimertinib in combination with anlotinib showed encouraging anti-tumor activity with an acceptable toxicity profile. Further randomized controlled trials are needed to assess the value of adding anlotinib to osimertinib in the first-line setting compared with standard-of-care osimertinib monotherapy.

Necitumumab is a monoclonal antibody against EGFR. It was initially tested in combination with cisplatin and gemcitabine as a first-line treatment for squamous NSCLC. The study showed 1.6 months OS benefit at the cost of increased toxicity [30]. It was hypothesized that duo blockade of EGFR by the combination of necitumumab and osimertinib may overcome EGFR-TKI resistance [31]. This combination was tested in a phase I dose escalation and expansion trial (NCT02496663). The trial recruited 101 EGFR-mutant NSCLC patients who developed resistance to first/second generation TKIs or osimertinib, or harbored EGFR ex20ins with disease progression on chemotherapy. The ORR among all patients was 19% (95% CI 11–27%). Grade 3 or higher TRAEs were seen in 38% patients, most commonly rash (21%) [31]. Necitumumab in combination with osimertinib is being tested in a phase II platform study (ORCHARD) for patients with EGFR-mutant NSCLC who had cancer progression on osimertinib. The trial is recruiting and estimated to complete in 2025.

Aurora kinases are enzymes within the serine/threonine kinase family and play key roles in mammalian cell mitosis and meiosis [32]. Preclinical studies have shown that aurora kinases are responsible for EMT-associated EGFR-TKI resistance [33,34]. PI3K/AKT/mTOR is a downstream pathway of EGFR. Activation of this pathway is reported to be a mechanism of EGFR-TKI resistance [35]. Adding an aurora kinase inhibitor or a mTOR inhibitor to osimertinib was tested in a phase I clinical trial (NCT04479306). A total of 40 patients with EGFR-mutant NSCLC who had cancer progression on osimertinib were assigned to two groups, and received osimertinib in combination with either alisertib, an aurora kinase inhibitor, or sapanisertib, an mTOR inhibitor. The osimertinib + sapanisertib arm showed ORR of 12.5%, DCR of 68.7%, and median PFS of 4.6 months; whereas the osimertinib + alisertib arm failed to show meaningful clinical efficacy [36]. The treatment was well tolerated. Grade 3 treatment emergent adverse events (TEAEs) occurred in 10% patients, most commonly hyperglycemia (45%) and stomatitis (40%). No grade 4 TEAEs were observed [36]. Further trials are needed to validate the clinical benefit of adding mTOR inhibitor to EGFR TKI.

A strategy of combining duo-EGFR blockade with HER2 blockade is being tested in a phase Ib/II trial. In the phase Ib dose escalation arm, 15 patients were treated with a triple therapy of osimertinib, necitumumab, and trastuzumab. The toxicities appear manageable (most commonly rash, headache, dry skin). It demonstrated a preliminary efficacy, as 5 of 10 evaluable patients achieved numerical reduction in tumor size [37]. The phase 2 arm is currently open for accrual.

Repotrectinib is a ROS1/TRK/ALK Inhibitor that was granted breakthrough therapy designation (BTD) for treating ROS1-postive or NTRK-positive NSCLC based on the results from TRIDENT-1 trial [38,39]. Additionally, as a pan-TRK inhibitor, repotrectinib can potentially overcome EGFR off-target resistance [39,40]. The combination of osimertinib and repotrectinib is being assessed in a phase I study (TOTEM trial). Eligible patients are required to have advanced EGFR-mutant NSCLC including those with brain metastasis or pre-treated with osimertinib [40]. The trial is conducted in Spain only. The trial is expected to finish recruitment by March 2023, and to be completed by June 2026 [40].

## 4. EGFR Exon 20 Insertions

EGFR exon 20 insertion (ex20ins) mutations, representing 4–12% of NSCLC EGFR mutations, are historically resistant to EGFR TKIs. In 2021, FDA approved amivantamab and mobocertinib for the treatment of patients with NSCLC who harbor EGFR exon 20 insertion mutations and have disease progression on or after platinum-based chemotherapy. The reported ORR and median PFS were 40% and 8.3 months for amivantamab, as well as 28% and 7.3 months for mobocertinib [41,42]. However, both drugs have significant toxicity (grade 3 or higher TRAEs 35–50%), and their effectiveness against CNS metastasis has not been established.

Sunvozertinib is a selective EGFR ex20ins inhibitor with weak activity against wild-type EGFR [43]. Its clinical efficacy is evaluated in the phase 1 WU-KONG1 and WU-KONG2 trials that contained 119 evaluable patients with EGFR ex20ins who had disease progression after platinum-based chemotherapy. The updated results were presented at WCLC 2022. In the RP2D 300 mg daily group, sunvozertinib achieved a prominent ORR of 52.4%, and the ORR in patients with brain metastasis remained high at 44% [44]. The toxicity appears manageable. Grade 3 or higher TRAEs most commonly were CPK elevation (11.2%), diarrhea (6.5%), and anemia (3%) [44]. The RP2D 300 mg daily dosing is further being tested in a phase II trial (WU-KONG6). The preliminary result was presented at ESMO 2022 (45). The clinical efficacy was similar to that in the phase I trial, with an ORR of 59.8% in all evaluable patients and 48.4% in patients with brain metastasis. The toxicity profile was similar to other EGFR TKIs [45]. Notably, 34% patients were pre-treated with PD-1/PD-L1 inhibitors. It would be interesting to know the response rate in the subgroup, as EGFR-mutant NSCLC patients who received immunotherapy initially usually developed severe AEs on subsequent EGFR TKIs [46,47]. Sunvozertinib appears to have an impressive efficacy in NSCLC with EGFR ex20ins as a subsequent systemic therapy, although this trend needs to be proved in larger double blind, controlled clinical trials. Additionally, it will be important to examine the effectiveness of sunvozertinib as first-line therapy for advanced NSCLC patients with EGFR ex20ins.

CLN-081 is a new EGFR TKI selectively against ex20ins rather than wild-type EGFR [48]. Its clinical application is being evaluated in an ongoing phase I/IIa trial (NCT04036682). The trial enrolled 73 patients with EGFR ex20ins NSCLC. A total of 66% of patients received 2 or more prior lines of therapy. A total of 38% patients had a history of CNS metastasis at baseline [49]. At the optimal dose of 100 mg bid, ORR was encouraging 41% and median PFS was 12 months. A total of 2 of 3 patients who had CNS target lesions achieved partial response or stable disease for more than a year. Most common grade 3 or higher TRAEs were anemia (10%), increased ALT (4%), and increased AST (4%) [49]. Compared to the two FDA-approved ex20ins inhibitors, CLN-081 appears to have similar or better clinical efficacy with potential effect on CNS metastasis, and a more tolerated toxicity profile. Data from further larger trials are awaited, especially for those patients with CNS involvement.

BLU-451 is another new inhibitor of EGFR ex20ins and has activity against both typical and atypical EGFR mutations on exon 20 and 21. It was shown to be CNS-penetrant, resulting in CNS tumor regression in a xenograft lung cancer mouse model [50]. It is being tested in a phase I/II trial (NCT05241873). Eligible patients include those with EGFT ex20ins NSCLC who have been treated with platinum-based chemotherapy with or without an EGFR ex20ins-targeted agent. Active asymptomatic brain metastasis are permitted in special cohorts [51].

## 5. ALK Rearrangements

Anaplastic lymphoma kinase (ALK) account for 3–7% of NSCLC patients [52,53]. Currently the FDA has approved five ALK inhibitors (ALKi) for NSCLC: crizotinib, ceritinib, alectinib, brigatinib, and lorlatinib. Treatment options after resistance to ALK inhibitors are limited.

SAF-189s is a novel next-generation ALK inhibitor with CNS penetration. Preclinical studies showed it can overcome most known resistance mutations of ALK [54]. It is being evaluated in a first-in-human phase I/II study in China (NCT04237805). The phase I data from 45 patients showed median PFS was 33.1 and 22.1 months (95% CI: 6.9–not reached and 13.8–26.6) in ALKi-naive and ALKi-pretreated patients, respectively. Most patients in phase II were ALKi-naive (*n* = 104, 69%), and the ORR was 92.3% and 65.4% (95% CI: 85.4–96.6 and 44.3–82.8) in ALKi-naive and ALKi-pretreated patients, respectively [54]. PFS data are not mature in phase II study yet. Common grade 3 or higher TRAEs were hyperglycemia (7%), hypertension (6%), and diarrhea (3%) [54]. The final results from this trial will help define the role of SAF-189s in ALK-altered NSCLC patients, particularly those who developed resistance to current ALK inhibitors.

APG-2449 is a TKI against ALK, ROS1, and focal adhesion kinase (FAK). It has been shown to have anti-tumor activity in ALK/ROS1-positive NSCLC mouse model [55]. It is being tested in a phase I dose escalation and expansion trial (NCT03917043) that enrolled 84 patients with ALK/ROS1 + NSCLC [56]. A total of 33% of them had received three or more lines of therapies. At the RP2D, it showed a good response rate in subsequent line setting (4 of 14 ALK+ patients resistant to second-generation TKIs achieved PR). The overall response rate was much higher in the first-line setting (80%, 8 of 10 patients). Four of eight patients with brain metastasis achieved objective response. Grade 3 or higher TRAEs only occurred in 7.1% patients [56]. APG-2449 appears to have a promising clinical efficacy with a favorable safety profile, although both need to be tested in further larger clinical trials.

## 6. ROS1

ROS1 rearrangements account for 1–2% of NSCLC. The ROS1 gene encodes a receptor tyrosine kinase belongs to the insulin receptor family [57]. Its fusion with other partner genes results in constant auto-phosphorylation and activates the downstream MAPK pathway that drive cancer cell proliferation [57,58]. The FDA has approved crizotinib and entrectinib for the treatment of ROS1-positive NSCLC. However, treatment resistance and disease progression inevitably occur in most patients [58]. In the TRIDENT-1 trial, repotrectinib demonstrated an ORR ranging from 40% to 67% in ROS1-positive NSCLC patients who had received one or more prior ROS1 TKI [39]. Taletrectinib, another ROS1 inhibitor, achieved an ORR of 90% in ROS1-positive, TKI-naïve, NSCLC patients in a phase II trial (NCT04395677) [59]. Both repotrectinib and taletrectinib were granted breakthrough therapy designation (BTD) by the FDA in 2022.

NVL-520 is a brain-penetrant, highly selective ROS1 inhibitor that is against ROS1 alterations including treatment-resistant G2032R solvent front mutation in preclinical studies [60]. ARROS-1 is a phase I/II trial (NCT05118789) designed to examine the pharmacokinetics, RP2D, safety, and preliminary clinical efficacy of NVL-520 in ROS1-positive solid tumors [61]. Patients are required to have received at least one ROS1 TKI therapy to be eligible for the trial. Patients with CNS metastasis are allowed. A most recent update was presented at the 34th EORTC-NCI-AACR (ENA) Symposium in Spain [62]. A total of 35 patients have enrolled and 34 of them have ROS1-positive NSCLC. Patients were heavily pre-treated, of which 71% had received two or more ROS1 TKIs. Among 21 evaluable patients, NVL-520 demonstrated an ORR of 48%, with no dose-limiting toxicities (DLTs) or treatment-related serious adverse events (SAEs) reported. In a smaller cohort of patients with CNS metastasis, the ORR was 73% (8/11). In patients with ROS1 G2032R-mutant cancers, 71.4% (5/7) achieved partial response [62]. The trial is continuing to recruit for the phase I part and the RP2D is yet to be determined.

## 7. KRAS

KRAS mutations had traditionally remained a pharmaceutical challenge because it does not have a deep binding pocket to fit in small molecule inhibitors [63]. The situation was changed in 2021 when FDA approved sotorasib, a first KRAS G12C inhibitor, for the treatment of locally advanced or metastatic KRAS G12C-mutant NSCLC patients who received at least one line of systemic therapy. In a single-arm phase II trial (CodeBreaK 100), sotorasib showed an ORR of 37.1% and a median PFS of 6.8 months [64]. Adagrasib is the second G12C inhibitor that showed clinical benefit with an ORR of 42.9% and a median PFS of 8.5 months in the second line setting [65].

GDC-6036 is an oral, irreversible, selective KRAS G12C inhibitor. It is more potent and selective to inhibit G12C mutation than sotorasib and adagrasib in vitro [66]. A phase I dose-escalation and dose-expansion trial (NCT04449874) evaluated GDC-6036 in patients with solid tumors harboring KRAS G12C mutation. The study included 59 NSCLC patients who received at least one line of systemic therapy and were naïve to G12C inhibitors. GDC-6036 as monotherapy showed an unconfirmed overall response rate (ORR) of 53% (30 of 57 evaluable patients). Grade 3 or higher TRAEs occurred in 16.9% patients, most commonly ALT elevation (6.8%), AST elevation (5.1%), and diarrhea (3.4%). No dose-limiting toxicities were reported [67]. GDC-6036 as monotherapy showed a preliminary promising clinical efficacy with tolerable toxicity profile for NSCLC patients carrying KRAS G12C in the second line setting. A phase II/III study including a docetaxel control arm is currently recruiting NSCLC patients (NCT03178552). The results are awaited to see if similar outcomes are reproducible.

D-1553 is another oral KRAS inhibitor that selectively and irreversibly binds KRAS G12C mutated protein [68]. In a phase I dose escalation and expansion trial, 79 NSCLC patients carrying KRAS G12C who failed at least one prior line of systemic therapy were given D-1553. The ORR was 37.8% and the median PFS was 7.6 months [69]. The data were encouraging and still immature given the high censor rate (68%). Grade 3 or above TRAEs occurred in 35.4% patients, mainly liver enzyme elevation [69]. A phase II trial is currently recruiting (NCT05383898).

## 8. RAF/MEK

MAPK pathway is commonly upregulated in cancer, which is manifested by increased activity of RAS, RAF, MEK, ERK. BRAF V600E mutation occurs in 1–2% patients with lung adenocarcinoma and results in constitutive activation of downstream MEK/ERK signaling, leading to cancer cell proliferation [70]. The combination of a BRAF inhibitor, dabrafenib, and a MEK inhibitor, trametinib, was shown to be effective against BRAF V600E-mutant NSCLC in a phase II trial. It demonstrated a ORR of 64% and median PFS of 10.8 months in the treatment-naïve group and a similar efficacy in the pretreated group [71,72]. The FDA approved dabrafenib and trametinib for the treatment of BRAF V600E-mutant metastatic NSCLC in 2017.

VS-6766 is a RAF/MEK clamp that inhibits both RAF and MEK and therefore prevents compensatory activation of RAF-dependent MEK phosphorylation [73]. VS-6766 monotherapy was shown objective responses in KRAS mutant NSCLC patients in a phase I trial [74]. Focal adhesion kinase (FAK) activation is a resistance mechanism of RAF and MEK inhibition. Defactinib, a FAK inhibitor, is being tested in combination with VS-6766 in the phase I FRAME trial (NCT03875820). This combination was well tolerated and had 15% ORR and 65% DCR [75]. A phase II study (RAMP 202, NCT04620330) is being conducted to evaluate the efficacy and safety of VS-6766 monotherapy and VS-6766 in combination with defactinib in NSCLC. This trial is open for enrollment of patients with KRAS or BRAF mutant NSCLC who have received at least one prior systemic therapy [76].

RAMP 203 is a phase I/II trial to assess the safety and efficacy of VS-6766 in combination with sotorasib. It is currently open for enrollment of patients with KRAS G12C mutant NSCLC. Eligible patients are required to be G12C inhibitors-naïve (cohort 1), or have disease progression while receiving G12C inhibitors (cohort 2) [77].

Crosstalks between RAF-MEK-ERK and PI3K-AKT-mTOR pathways are well established in tumorigenesis [78]. A combination of VS-6766 and everolimus, a mTOR inhibitor, is being evaluated in the aforementioned phase I trial (NCT02407509) to evaluate its safety and MTD. A total of 28 patients with RAS or RAF mutant cancers were included in the study and the median lines of previous treatment was 3 (range 0–7). At the recommended phase II dose, Grade 3 or above TRAEs were rash (18%) and pruritus (7%). In the KRAS mutant NSCLC expansion cohort, 2 of 10 patients showed objective response. The KRAS mutation was G12D in these two patients, and PFS was 35.8 and 41.8 months with treatment ongoing. The median PFS was 6.35 months (95% CI 3.52–not reached), a promising result in this heavily pre-treated cohort [79]. The combination of VS-6766 and everolimus is tolerable and has an encouraging preliminary clinical efficacy across different KRAS mutation variants. Further phase II/III trials are needed to prove its effectiveness in this subgroup of NSCLC patients who have KRAS mutations other than G12C and are lack of available targeted therapies.

## 9. ERK 1/2

ERK is a downstream effector in the RAF-MEK-ERK pathway. Inhibition of ERK may overcome resistance mechanisms of BRAF and MEK inhibitors [80]. ASTX029 is a potent, selective dual-mechanism ERK inhibitor that inhibits both ERK catalytic activity and the phosphorylation of ERK itself by MEK, despite not directly inhibiting MEK activity [81]. It demonstrated anti-tumor activity previously in xenograft mouse models [81]. It is being evaluated in a phase I clinical trial including 76 patients who had relapsed/refractory solid tumors (NCT03520075). Adverse events were similar to those previously reported with MEK inhibitors. Grade 3 or above TRAEs included diarrhea (*n* = 1), rash (*n* = 1), and malaise (*n* = 1). A dose level of 200 mg daily continuously was selected for investigation in the phase II study [82]. Among 12 patients who had NSCLC, 3 of them achieved durable partial response. Interestingly, all three of them carried KRAS mutation (G12A, G12D, G12V) [82]. The results from phase II trial will help assess its efficacy in patients with MAPK-altered solid tumors, especially in NSCLC patients with KRAS mutations other than G12C.

## 10. HER2

HER2-mutant NSCLC is a rare subgroup (2–4%) of NSCLC. It is associated with poor outcomes and short survival compared with other subtypes of NSCLC. There is an unmet need for developing targeted therapy in this subgroup of patients. Recently, the FDA granted accelerated approval for fam-trastuzumab deruxtecan-nxki (T-DXd) as a second or further line of therapy for patients with HER2-mutant NSCLC, based on DESTINY-Lung01 and DESTINY-Lung02 trials. T-DXd showed promising ORR of 58% and median DOR of 8.7 months in this pre-treated subpopulation [83,84]. Drug-related interstitial lung disease, a notable TRAE, occurred less frequently in patients treated with a lower dose (5.9% vs. 14.0%, in dosing groups 5.4 mg/kg vs. 6.5 mg/kg) [83,84]. A phase III trial, DESTINY-Lung04, is currently evaluating the efficacy and safety of T-DXd in the first-line setting for the treatment of HER2-mutant NSCLC. Another HER-2 targeted agent, BI 1810631, selectively inhibits HER2 but spares wild-type EGFR, thus limiting the toxicities associated with off-target EGFR inhibition [85]. A phase Ia/Ib trial (NCT04886804) is open to recruit approximately 96 patients worldwide who have HER2+ advanced solid tumor, including about 30 patients who have HER2 ex20ins mutant, pretreated, advanced NSCLC in the Ib arm. The primary endpoints are MTD in phase Ia and ORR in phase Ib [85,86].

## 11. MET

The mesenchymal-epithelial transition (MET) gene is a proto-oncogene that regulates cell proliferation, cell adhesion, and angiogenesis [87]. MET genomic alterations occur in about 4% of patients with NSCLC [52]. The alterations consist of exon 14 skipping mutations, MET amplification, and MET fusions [88]. Capmatinib and tepotinib are the only two FDA-approved agents for patients with metastatic NSCLC harboring MET exon 14 skipping mutations, based on two phase II trials. The ORR was 68% for capmatinib and 43% for tepotinib in the treatment-naïve population [89,90].

Telisotuzumab vedotin is an antibody-drug conjugate consisting of a c-Met antibody and a microtubule inhibitor. It is being evaluated in a phase II trial including patients with advanced c-MET-overexpressing NSCLC who had received less than two prior therapies. The ORR was 36.5% in the non-squamous EGFR wild-type cohort, but was less in the non-squamous EGFR-mutant and squamous cohorts [91]. Two patients developed grade 5 AEs (one sudden death and one pneumonitis, possibly related) raised a safety concern [91].

REGN5093 is a human bispecific antibody that binds to two distinct epitopes of MET and causes internalization and degradation of MET [92]. REGN5093-M114 is an antibody drug conjugate (ADC) that combines this bispecific antibody to a microtubule inhibitor. A phase I/II trial (NCT04982224) is open for enrollment to evaluate its safety, tolerability, recommended dosing, and preliminary antitumor activity [93]. Eligible patients need to have MET-overexpressing NSCLC and no further approved therapies are available.

## 12. AXL

AXL is a receptor tyrosine kinase that is known to be associated with treatment resistance in NSCLC [94,95]. BGB324 (bemcentinib), a selective oral AXL inhibitor, has shown synergistic anti-tumor activity with immune checkpoint inhibitor by overcoming immune suppression conferred by STK11 mutation in a NSCLC xenograft mouse model (96). In a phase II trial (NCT03184571) testing the combination of bemcentinib and pembrolizumab in advanced NSCLC patients, three of those were found to have STK11 mutations, and all of them demonstrated objective response [96]. In November 2021, the FDA granted a fast track designation to bemcentinib in combination with a PD-1/PD-L1 agent in the treatment of patients with STK11-altered advanced or metastatic NSCLC without actionable mutations. Given the promising results on subsequent lines of therapy, on 11 October 2022, a new phase Ib/IIa trial (NCT05469178) was initiated to test the combination of bemcentinib and chemo-immunotherapy in the first-line setting for the treatment of STK11 mutant NSCLC patients.

Additionally, bemcentinib was also shown synergistic anti-tumor activity with docetaxel in in vivo studies [97]. A phase I dose escalation and expansion trial (NCT02922777) is currently evaluating the combination of bemcentinib and docetaxel in patients with advanced NSCLC. The trial enrolled 21 patients who were heavily pre-treated (median number of prior therapy was 3, range 1–13). A total of 27% (4 of 15) patients had partial response, and 60% (9 of 15) patients had stable disease. Notably, 76% patient had grade 3 or above neutropenia, albeit received prophylactic G-CSF support [97].

## 13. IL1RAP

IL-1 dysregulation is implicated in tumorigenesis, tumor progression, and resistance to chemotherapy [98]. Interleukin-1 Receptor Accessory Protein (IL1RAP) is a co-receptor of IL-1R and is required for IL-1 signaling [99]. Nadunolimab (CAN04) is a humanized antibody targeting IL1RAP that has a synergetic anti-tumor effect in combination with platinum-based chemotherapy in an in vitro model [99]. Nadunolimab was evaluated in combination with chemotherapy in a phase I/IIa trial CANFOUR (NCT03267316). A total of 33 patients with advanced NSCLC received nadunolimab along with cisplatin and gemcitabine as first-line therapy or after progression on pembrolizumab. On an interim analysis, the ORR was 53% and the PFS was 6.8 months. TRAEs of grade 3 or higher included neutropenia (58%), febrile neutropenia (9%), thrombocytopenia (30%), and anemia (18%) [100]. In this study, nadunolimab combined with chemotherapy demonstrated clinical efficacy and the toxic profile was expected. However, the trial did not choose more commonly used chemo regimens for NSCLC such as pemetrexed or paclitaxel. Further studies are also needed to compare its clinical benefit to the current standard-of-care chemoimmunotherapy.

## 14. NRF2

NRF2 (encoded by NFE2L2) is a transcription factor that regulates oxidative stress response. It is negatively regulated by KEAP1. Genetic alterations to NFE2L2 and KEAP1 lead to constitutive activation of NRF2, that occurs in 30% of lung squamous cell carcinoma and 25% of lung adenocarcinoma [101,102,103]. Telaglenastat (CB-839) is a glutaminase inhibitor that has anti-tumor activity in KEAP1/NRF2-mutated NSCLC cell lines and xenograft models [104]. Sapanisertib, a mTOR inhibitor, was shown to have selective anti-tumor activity in NRF2-activating NSCLC in an in vivo xenograft model [105]. Therefore, the combination of telaglenastat and sapanisertib was hypothesized to have synergistic efficacy in treating NRF2/KEAP1-altered NSCLC [106]. This combination is being tested in a phase I trial (NCT04250545). A total of 13 patients were enrolled in the dose escalation portion, and the primary endpoint of identifying the recommended expansion dose (RED) was reached. Most patients achieved tumor shrinkage (5/8). This combination appears safe and tolerable at the RED [106]. The expansion portion of the study contains four cohorts suitable for patients with different KRAS or NFE2L2/KEAP1 mutation status. A separate phase 2 trial (NCT02417701) is also assessing the potential role of sapanisertib in patients with stage IV or recurrent LSCC.

## 15. ACSS2

**Acyl-coenzyme** A synthetase short-chain family member 2 (ACSS2) is an enzyme responsible for acetyl-CoA synthesis. Its gene expression promotes the growth of several human solid tumors [107]. MTB-9655, the first oral inhibitor of ACSS2, was shown to have anti-tumor effect in xenograft mouse model of ACSS2-high lung cancer [108]. In a phase I dose escalation and expansion trial (NCT04990739), 10 patients who had advanced solid tumors unamendable to standard therapies received this drug and demonstrated few toxicities. MTD has not been reached yet and the dose escalation is continuing [108].

## 16. FGFR

Fibroblast growth factor receptor (FGFR) alterations contribute to oncogenesis in multiple tumor types. Pemigatinib, a selective FGFR1-3 inhibitor, was showing response across several tumor types including NSCLC in the phase I FIGHT-101 study [109]. FIGHT-210 is a phase II single-arm multicenter trial aiming to recruit 125 patients with advanced NSCLC with FGFR1-3 alterations who progress on available therapies. It is currently recruiting. Futibatinib is an irreversible FGFR1-4 inhibitor, which demonstrates antitumor response in several solid tumors carrying FGFR aberrations [110]. Preclinical studies showed that futibatinib in combination with a MEK inhibitor had synergistic antitumor effect in KRAS-mutant NSCLC cell lines. A phase I/II open-label trial (NCT04965818) is being conducted to assess the safety and preliminary efficacy of futibatinib and binimetinib (MEK inhibitor) in patients with advanced NSCLC with KRAS mutation [111]. The trial is still ongoing, but has completed recruitment. Rogaratinib, a pan-FGFR inhibitor, was studied in patients with advanced and pretreated lung squamous cell carcinoma in the phase II SAKK 19/18 trial. It enrolled 15 patients with FGFR1-3 mRNA overexpression. However, the trial was closed prematurely due to futility observed from its initial results. Median PFS was 1.6 months, with median OS being 3.5 months. The majority of participants, 60%, discontinued the drug due to disease progression. Among all of them, 46.7% had stable disease and 33% showed disease progression [112].

## 17. Squamous Cell Carcinoma

As mentioned above, LSCC is the second-most common histologic type of NSCLC, accounting for approximately 20% of cases of lung cancer in the USA [113]. While many advancements with targeted therapy had positively impacted outcomes in patients with adenocarcinoma of the lung, the same has not been seen with LSCC, as the most common driver mutations seen on adenocarcinoma are infrequently observed in LSCC [114,115]. Despite that, LSCC still presents significant gene mutations affecting a diversity of cellular pathways that may be potential targets for cancer therapy [116]. This subtype of NSCLC is an active subject of ongoing research aiming at recognizing specific mutations and pharmacologic mechanisms to target them. Table 3 summarizes the trials presented below.

## 18. FOLR1/PSMA

Folate-receptor 1 (FOLR1) and prostate specific membrane antigen (PSMA) are overexpressed in solid tumors including lung cancer, prostate cancer, and renal cell carcinoma [117]. They are present both on tumor cells and on endothelial cells. CBP-1018 is the first bi-ligand drug conjugate targeting both FOLR1 and PSMA, with a monomethyl auristatin E drug payload [118]. A phase IA/IB open-label trial (NCT04928612) is currently enrolling patients that have relapsed after prior standard lines of therapy, with four different cohorts. One of them is comprised by patients with advanced LSCC. The primary objectives are safety, tolerability, dose limiting toxicity, and MTD assessment. Preliminary ORR, PFS, and duration of response data will also be assessed.

## 19. ATR

Ataxia telangiectasia and Rad3-related protein kinase (ATR) is one of the regulators of DNA damage response. It is potential target especially for cells that have lost ataxia-telangiectasia-mutated kinase (ATM) or p53. In this way, berzosertib has been studied in combination with DNA-damaging drugs. This compound is a first-in-calls inhibitor of ATR, with observed antitumor activity in preclinical studies [119]. A phase 1 study of bersozertib combined with gemcitabine with or without cisplatin in patients with advanced solid tumors (12% of patients with NSCLC) showed that this regimen was safe and well-tolerated [120]. When combined with gemcitabine alone, it led to partial response in 8.3% of patients, with 60.4% experiencing stable disease. In the group of berzosertib with gemcitabine and cisplatin, 14% experienced partial response, with 57% showing stable disease.

Berzosertib is now being studied in a phase IB and randomized open-label phase II study combining it with carboplatin, gemcitabine, and pembrolizumab in patients with LSCC that were never treated with chemotherapy (NCT04216316). Patients are currently being enrolled.

## 20. NSD3

NSD3 is a histone methyltransferase located on chromosomal region 8p 11–12. It has been demonstrated to participate in the tumorigenesis of LSCC. By depleting NSD3 from animal models with patient-derived xenografts, researchers have been able to slow down tumor growth in mice [57,121]. This study also demonstrated that LSCC with NSD3 may render cancer cells susceptible to bromodomain and extraterminal (BET) inhibitors. Similar effects have been already documented in acute myeloid leukemia [9,122]. As no NSD3 inhibitors are currently available, investigators started considering the role of BET inhibitors in this setting. A phase II trial of ZEN003694, a BET inhibitor, is currently enrolling patients with recurrent or metastatic LSCC with NSD3 amplification (NCT05607108).

## 21. PI3K Pathway

The phosphatidylinositol 3-kinase (PI3K)/ protein kinase B (AKT)/ mammalian target of rapamycin (mTOR) pathway is essential in cell cycle regulation and survival [123]. It is also commonly involved in the genesis of cancer cells, as it directly affects cell proliferation and apoptosis. As such, inhibitors of this pathway have been studied as potential anti-cancer drugs. Some have been approved, being potential therapeutic lines in chronic lymphocytic leukemia and follicular lymphoma, for example.

Copanlisib is a pan-class I PI3K inhibitor that has been studied in combination with nivolumab, a PD-1 inhibitor, in a phase IB trial (NCT03735628). The combination was used in patients with advanced solid tumors. It was demonstrated to be safe and well tolerated. Within the treated group, progression of disease was seen in 25% of patients, with the remaining 75% demonstrating some degree of disease control [124]. Results for the phase II of this trial have not yet been published.

Other combination strategies involving a PI3K inhibitor are also being assessed. Its use in association with a cyclin-dependent kinase 4 and 6 (CDK4/6) inhibitors is being considered in a phase I trial (NCT03065062). This trial is employing palbociclib, a CDK4/6 inhibitor, in combination with gedatolisib, a PI3K/mTOR inhibitor, in patients with advanced LSCC and other solid tumors. CDK4/6 inhibitors are known to lead to cell cycle arrest, but are also found to indirectly affect other intracellular pathways. This mechanism has been described in breast cancer, the most common clinical indication that employs CDK4/6 inhibitors, but also in other malignancies such as pleural mesothelioma [125,126].

## 22. Conclusions

Targeted therapy has achieved tremendous success and revolutionized the treatment landscape of NSCLC. In advanced disease, however, treatment resistance and failure eventually occur in majority of patients, which remains the main challenge for NSCLC targeted treatment. This challenge is much attributed to the complexity of tumor biology including intratumor heterogeneity, cancer genome evolution, and resistance mutations due to selective pressure imposed by current therapy [127,128,129]. The development of more effective targeted therapies relies on a better understanding of NSCLC at the genome, transcriptome, and proteome levels [5,130]. New discovery of oncogenic driver alterations and treatment-resistant mechanisms are being transformed into new drug developments and being tested in early phase clinical trials. We are confident that targeted therapy continues to be an ever-changing field in NSCLC treatment, with a goal to achieve individualized precision medicine that further improves patients’ overall outcome.

## Figures and Tables

**Table 1:** Recent first-in-human phase I/II clinical trials of EGFR targeted agents for NSCLC.

Drug Name	Mechanism of Action	Clinical Trial	Phase	Study Design	Target Group	Status	Preliminary Result
Lazertinib	EGFR TKI	NCT03046992	I/II	Monotherapy	EGFR T790M+ NSCLC progressed after TKI	Active, not recruiting	*n* = 127, ORR 54%
		NCT03046992, additional cohort C	I/II	Monotherapy	First-line therapy in EGFRm + NSCLC	Active, not recruiting	*n* = 43, ORR 70%, mPFS 24.6mo
		NCT04248829 (LASER301)	III	Monotherapy	First-line treatment in advanced EGFRm + NSCLC	Active, not recruiting	
Amivantamab	EGFR-MET bispecific antibody	NCT04077463 (CHRYSALIS-2)	I	Combination with lazertnib	EGFRm+ NSCLC progressed after TKI	Recruiting	*n* = 116, ORR 32%
		NCT02609776 (CHRYSALIS)	I	Combination with lazertnib	First-line treatment in advanced EGFRm + NSCLC	Recruiting	*n* = 20, ORR 100%
		NCT04487080 (MARIPOSA)	III	Combination with lazertnib	First-line treatment in advanced EGFRm + NSCLC	Active, not recruiting	
Aumolertinib	Third generation EGFR TKI	NCT02981108 (APOLLO)	I/II	Monotherapy	EGFR T790M + NSCLC progressed after TKI	Active, not recruiting	*n* = 244, ORR 68.9%, mPFS 12.4mo
		ChiCTR2100044216	I/II	Combination with icotinib	First-line therapy in EGFRm + NSCLC with brain metastasis		*n* = 12, ORR 100%
BLU-945	EGFR TKI	NCT04862780 (SYMPHONY)	I/II	Monotherapy, or combination with osimertinib	EGFRm + NSCLC progressed after TKI	Recruiting	
BBT-176	Fourth generation EGFR TKI against C797S mutation	NCT04820023	I/II	Monotherapy	EGFRm + NSCLC progressed after TKI	Recruiting	
BLU-701	EGFR TKI against C797S mutation	NCT05153408 (HARMONY)	I/II	Monotherapy, or combination with osimertinib or chemotherapy	EGFRm + NSCLC progressed after TKI	Recruiting	
Necitumumab	EGFR monoclonal antibody	NCT02496663	I	Combination with osimertinib	EGFRm + NSCLC progressed after TKI	Active, not recruiting	*n* = 101, ORR 19%
Sunvozertinib	EGFR ex20ins inhibitor	NCT03974022 and CTR20192097 (WU-KONG1 and 2)	I/II	Monotherapy	EGFR ex20ins + NSCLC progressed after platinum-based chemotherapy	Recruiting	*n* = 119, ORR 52.4%
		CTR20211009 (WU-KONG6)	II	Monotherapy	EGFR ex20ins + NSCLC progressed after platinum-based chemotherapy		*n* = 97, ORR 59.8%
CLN-081	EGFR ex20ins inhibitor	NCT04036682	I/II	Monotherapy	EGFR ex20ins + NSCLC progressed after prior therapy	Recruiting	At optimal dose, *n* = 39, ORR 41%, mPFS 12mo
BLU-451	EGFR ex20ins inhibitor	NCT05241873	I/II	Monotherapy	EGFR ex20ins + NSCLC progressed after platinum-based chemotherapy	Recruiting	

**Table 2:** Recent first-in-human phase I/II clinical trials of non-EGFR targeted agents for NSCLC.

Drug Name	Mechanism of Action	Clinical Trial	Phase	Study Design	Target Group	Status	Preliminary Result
Patritumab deruxtecan	Anti-HER3 antibody-drug conjugate	NCT03260491	I	Monotherapy	EGFRm + NSCLC progressed after TKI	Recruiting	*n* = 57, ORR 39%, mPFS 8.2mo
		NCT03260491, cohort 2	I	Monotherapy	EGFR wild-type NSCLC progressed after chemotherapy +/− immunotherapy	Recruiting	*n* = 47, ORR 28%, mPFS 5.4mo
		NCT04619004 (HERTHENA-Lung01)	II	Monotherapy	EGFRm + NSCLC progressed after TKI	Recruiting	
Anlotinib	Multi-targeting TKI	NCT04770688	I/II	Combination with osimertinib	First-line therapy in EGFRm + NSCLC	Recruiting	*n* = 25, ORR 65.2%, DCR 95.7%
Alisertib	Aurora kinase inhibitor	NCT04479306	I	Combination with osimertinib	EGFRm + NSCLC progressed after TKI	Active, not recruiting	No meaningful clinical efficacy
Sapanisertib	mTOR inhibitor	NCT04479306	I	Combination with osimertinib	EGFRm + NSCLC progressed after TKI	Active, not recruiting	ORR 12.5%, DCR 68.7%, mPFS 4.6mo
Necitumumab and trastuzumab	HER2 monoclonal antibody	NCT04285671	I/II	Combination with osimertinib	EGFRm + NSCLC progressed after osimertinib	Recruiting	5 of 10 patients achieved tumor size reduction
Repotrectinib	ROS1/TRK/ALK Inhibitor	NCT04772235	I	Combination with osimertinib	Advanced EGFRm+ NSCLC	Recruiting	
SAF-189s	ALK inhibitor	NCT04237805	I/II	Monotherapy	ALK-altered NSCLC with/without prior ALK inhibitor	Recruiting	ORR 92.3% and 65.4% in ALKi-naive and ALKi-pretreated, respectively
APG-2449	ALK/ROS1/FAK TKI	NCT03917043	I	Monotherapy	ALK-altered NSCLC with/without prior therapy	Recruiting	4 of 14 patients resistant to 2nd-gen TKIs achieved PR
Taletrectinib	ROS1 inhibitor	NCT04395677	II	Monotherapy	ROS1-positive, TKI-naïve, NSCLC	Recruiting	*n* = 40, ORR of 90%
NVL-520	ROS1 inhibitor	NCT05118789 (ARROS-1)	I/II	Monotherapy	ROS1-positive solid tumors, progressed after ROS1 TKI	Recruiting	*n* = 21, ORR of 48%
GDC-6036	KRAS G12C inhibitor	NCT04449874	I	Monotherapy	KRAS G12C + NSCLC, progressed after prior therapy	Recruiting	*n* = 57, ORR 53%
		NCT03178552 (BFAST), cohort G	II/III	Monotherapy	KRAS G12C + NSCLC, progressed after prior therapy	Recruiting	
D-1553	KRAS G12C inhibitor	NCT05383898	I/II	Monotherapy	KRAS G12C + NSCLC, progressed after prior therapy	Recruiting	*n* = 79, ORR 37.8%, mPFS 7.6mo
ASTX029	ERK inhibitor	NCT03520075	I/II	Monotherapy	advanced solid tumors refractory to available therapies	Recruiting	3 of 12 NSCLC patients achieved durable PR
VS-6766	Dual MEK and RAF inhibitor	NCT02407509	I	Monotherapy or combination with everolimus	Advanced solid tumors or multiple myeloma harboring RAS-RAF-MEK pathway mutations	Recruiting	In monotherapy cohort, 3 of 10 KRASm+ NSCLC achieved PR; in combination cohort, 2 patients with G12D+ had PFS 35.8mo and 41.8mo
		NCT03875820 (FRAME)	I	Combination with defactinib	RAS or RAF mutant advanced solid tumors refractory to conventional treatment	Recruiting	ORR 15%, DCR 65%
		NCT04620330 (RAMP 202)	III	Monotherapy or combination with defactinib	KRAS or BRAF mutant NSCLC progressed after prior therapy	Recruiting	
		NCT05074810 (RAMP 203)	I/II	Combination with sotoracib	KRAS G12C+ NSCLC with/without prior G12C inhibitors	Recruiting	
Trastuzumab deruxtecan (T-DXd)	Anti-HER2 antibody-drug conjugate	NCT05048797 (DESTINY-Lung04)	III	Monotherapy	First-line therapy for HER2m + advanced NSCLC	Recruiting	
BI 1810631	Selective HER2 inhibitor	NCT04886804	I	Monotherapy	HER2-altered advanced solid tumors progressed after available therapies	Recruiting	
Telisotuzumab vedotin	Anti-MET antibody-drug conjugate	NCT03539536 (LUMINOSITY)	II	Monotherapy	Advanced MET-overexpressing NSCLC received less than two prior therapies	Recruiting	*n* = 122, ORR 36.5% in non-squamous EGFR wild-type cohort
REGN5093-M114	Anti-MET antibody-drug conjugate	NCT04982224	I/II	Monotherapy	Advanced MET-overexpressing NSCLC progressed after current therapies	Recruiting	
BGB324 (bemcentinib)	Selective AXL inhibitor	NCT05469178	I/II	Combination of with chemo-immunotherapy	First-line therapy for non-squamous NSCLC with STK11 mutation	Not yet recruiting	
		NCT02922777	I	Combination with docetaxel	Advanced NSCLC progressed after prior therapy	Active, not recruiting	*n* = 21, PR 27%
Nadunolimab (CAN04)	Anti-1L1RAP antibody	NCT03267316 (CANFOUR)	I/II	Combination with cisplatin and gemcitabine	Advanced NSCLC treatment-naïve or progressed after pembrolizumab	Recruiting	*n* = 33, ORR 53%, PFS 6.8mo
Telaglenastat (CB-839)	Glutaminase inhibitor	NCT04250545	I	Combination with mTOR inhibitor sapanisertib	Advanced NSCLC progressed after prior therapy	Suspended (pending dose update)	5 of 8 patients had tumor shrinkage
MTB-9655	ACSS2 inhibitor	NCT04990739	I	Monotherapy	Advanced solid tumors failed standard treatment	Recruiting	
Pemigatinib	FGFR1-3 inhibitor	NCT05253807	II	Monotherapy	Advanced FGFR + NSCLC progressed after available therapies	Recruiting	
Futibatinib	FGFR1-4 inhibitor	NCT04965818	I/II	Combination with MEKi binimetinib	Advanced solid tumors failed standard treatment	Active, not re-cruiting	

**Table 3:** Summarizes recent first-in-human clinical trials of non-EGFR targeted agents for lung squamous cell carcinoma.

Drug Name	Mechanism of Action	Clinical Trial	Phase	Study Design	Target Group	Status	Preliminary Result
CBP-1018	FOLR1 and PSMA bi-ligand drug conjugate	NCT04928612	I	Monotherapy	LSCC and other solid tumor patients with relapse on prior lines of therapy	Recruiting	
Berzosertib	ATR inhibitor	NCT04216316	I/II	Combination with carboplatin, gemcitabine, and pembrolizumab	LSCC patients that are chemotherapy-naive	Recruiting	
Rogaritinib	Pan-FGFR inhibitor	NCT03762122	II	Monotherapy	Advanced and pretreated LSCC with FGFR1-3 mRNA overexpression	Terminated	mPFS 1.6mo mOS 3.5mo
ZEN003694	BET inhibitor	NCT05607108	II	Monotherapy	Recurrent or metastatic LSCC with NSD3 amplification	Recruiting	
Copanlisib	Pan-class I PI3K inhibitor	NCT03735628	I/II	Combination with nivolumab	Advanced solid tumors	Active, recruiting phase completed	Disease control rate: 75%
Gedatolisib	PI3K/mTOR inhibitor	NCT03065062	I	Combination with palbociclib	Advanced LSCC and other solid tumors	Recruiting	
